# Human Placenta-Derived Mesenchymal Stem Cells Ameliorate Diabetic Neuropathy via Wnt Signaling Pathway

**DOI:** 10.1155/2022/6897056

**Published:** 2022-09-06

**Authors:** Songsong Pan, Sushant S. Hada, Yang Liu, Chao Hu, Mengdie Zhou, Shaoqiu Zheng, Minjie Xu, Changsheng Shi, Shiwu Yin, Xiaoyun Xie

**Affiliations:** ^1^Department of Interventional and Vascular Surgery, Shanghai Tenth People's Hospital, Tongji University School of Medicine, Shanghai, China; ^2^Division of Geriatrics, Tongji Hospital, Tongji University, School of Medicine, Shanghai, China; ^3^Department of Interventional Therapy, The Third Affiliated Hospital of Wenzhou Medical University, Wenzhou, Zhejiang, China; ^4^Department of Interventional & Vascular Surgery, Hefei Second People's Hospital, Hefei Hospital Affiliated to Anhui Medical University, 1 Guangde Road, Hefei, Anhui Province 230011, China

## Abstract

**Objectives:**

To investigate the effect of placenta-derived mesenchymal stem cells (PMSCs) on diabetic peripheral neuropathy and explore the role of Wnt signaling pathway.

**Method:**

Twenty-seven male db/db mice were randomly categorized into the control group, PMSC group, and PMSC treatment with Wnt inhibitor treatment group. Intervention was initiated in week 22. Thermal stimulation response was determined with a plantar analgesia tester. The mice were sacrificed on 7, 14, and 28 days. The morphology of sciatic nerves was observed by electron microscopy, and the expression of protein gene product (PGP) 9.5, S100*β*, and Ku80 was detected by immunofluorescence. Bax, *β*-catenin, and dishevelled1 (DVL1) were detected by western blot.

**Results:**

Thermal stimulation response was improved in the PMSC group on 14 and 28 days. Compared with the control group, PGP9.5 was increased in the PMSC group, accompanied by a significant increase in the expression of S100*β*. On the contrary, LGK974 inhibited the effect of PMSCs on thermal stimulation response and the expression of PGP9.5 and S100*β*. Both PGP9.5 and S100*β* were correlated with Ku80 in fluorescence colocalization. The myelin sheath of sciatic nerves in the PMSC group was uniform and dense compared with that in the control group. The effects of PMSCs promoting myelin repair were significantly inhibited in the PMSC+LGK974 group. Bax in the PMSC group expressed less than the control group. In contrast, the expressions of *β*-catenin and DVL1 were higher compared with that in the control group on the 14th and 28th days. The expression of DVL1 and *β*-catenin was lower in the PMSC+LGK974 group than in the PMSC group.

**Conclusions:**

PMSCs improved the symptoms of diabetic peripheral neuropathy, along with the improvement of nerve myelin lesions, promotion of nerve regeneration, and activation of Schwann cells, which might be related to the regulation of Wnt signaling pathway and inhibition of apoptosis.

## 1. Introduction

Diabetes is spreading rapidly in the world. In the past 20 years, the incidence rate of diabetes increased from 150 million cases in 2000 to 425 million in 2017 [1], and it is estimated that by 2040, the incidence of diabetes will reach 642 million [2]. Among the complications of diabetes mellitus (DM), pain caused by neuropathy has the most significant impact on a patient's quality of life [3]. According to reports, 50% of diabetic patients suffer from neuropathy. Neuropathy is considered to be the earliest occurrence and most influential complication of diabetes. In the early stage of the disease, small sensory perception is usually affected. Electrophysiological functions have been almost unaffected, although some slowing down in nerve conduction speed has been observed [4, 5]. Symptoms of diabetic neuropathy depend on the type of damaged nerve. Enhanced function symptoms performed hypersensitivity (nonstimulating pain and increased pain sensitivity), while dysfunction symptoms were mainly manifested as tactile and thermal hyposensitivity, and in the advanced stages, patients may even develop complete loss of sensation [6]. Among the various neuropathic symptoms, cardiac autonomic neuropathy may result in sudden death, abnormal pain seriously affects the patient's comfort, and pain insensitivity leads to the high risk of burns, trauma and ulcers, eventually leading to amputation [7].

According to the American Diabetes Association, specific treatment for the underlying nerve damage, other than improved glycemic control, is currently not available [8]. Glycemic control can slow the progression of diabetic peripheral neuropathy and cardiac autonomic neuropathy in type 2 diabetes, but it does not reverse neuronal loss [9]. Neuropathic pain control relies on pharmaceutical interventions like Pregabalin and duloxetine [8]. Because intensive diabetes therapy completely prevents neither the development of diabetic neuropathy nor its progression and the efficacy of symptomatic treatments for neuropathic pain is limited, there is a continuing need for the development of novel treatments [10].

Stem cell therapy may become a novel strategy for diabetic neuropathy therapy [11]. Studies demonstrated that stem cell transplantation partially repaired damaged neurons and delayed the progress of amyloidosis in the treatment of Alzheimer's disease [8]. Stem cell treatment also promoted the repair of the injured plane in the treatment of transverse nerve injury caused by trauma [7]. Importantly, compared with other treatment options, stem cell transplantation can reverse neuropathy, which may be associated with high proliferation and differentiation of stem cells; also the specific mechanism remains unclear [12].

The Wnt signaling pathway can improve nerve injury and promote repair. *β*-Catenin and GSK3-*β* are considered to be key proteins in the repair of damage, and their expression levels are greatly affected by the inhibitors. Studies found that regulation of Wnt signaling pathway ameliorated neurodegenerative symptoms in elderly rats [13]. Previous reports have revealed that the inhibition of GSK3-*β* promoted the clearance of injured nerve fragments and accelerated nerve repair, which may have relation to the increased expression of *β*-catenin [14]. The expression of Wnt3a mRNA was conspicuously increased for 2 weeks after nerve injury and remained upregulated for 8 weeks. Additionally, 2 months after the injury, *β*-catenin was activated [15]. Therefore, we hypothesized that the Wnt signaling pathway might also play a pivotal role in treating PMSCs for diabetic neuropathy.

## 2. Materials and Methods

### 2.1. Animal Preparation and Groups

Adult male db/db mice aged 9 weeks with blood glucose of 28.18 ± 4.90 mmol/L and body weight of 48.04 ± 2.84 g were obtained from the Model Animal Research Center of Nanjing University (strain: BKS.Cg-Dock^7m+/+^Lepr^db/Nju^). The Animal Ethics Committee of Tongji University approved the protocol for mice. These were stored in Specific Pathogen Free (SPF) conditions. The ambient temperature of the rearing was 22°C-25°C. The relative humidity of the animal house was 50-60%, and mice were maintained at a 12 h light/12 h dark cycle with unrestricted access to food and water and 5 mice per cage.

**Figure 1:**
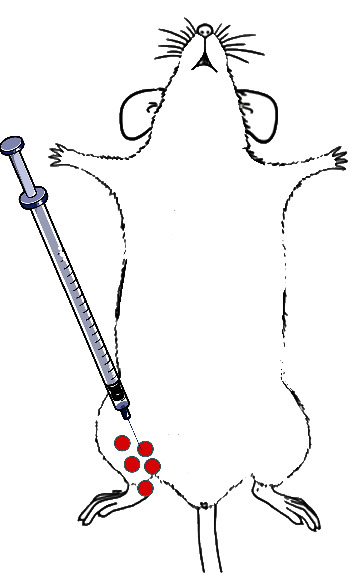
Diagram of PMSCs injection. PMSCs were injected at red dot.

Twenty-seven male db/db mice were randomly categorized into three groups: control group, PMSC treatment group (PMSC group), and PMSC treatment with Wnt inhibitor treatment group (PMSC+LGK974 group). BKS mice were used in comparison with db/db mice before treatment. Nerve injury was assessed by thermal pain. On the 22nd week, there was a significant difference between db/db mice and BKS mice. So at 22 weeks, the mice were injected with 10^6^ PMSCs at five different sites in limb (Figure 1, inject at the red dot), in the PMSC group and the PMSC+LGK974 group. LGK974 is an inhibitor of Wnt signaling pathway and is used to observe the therapeutic mechanism of PMSCs. And to show the effects of LGK974, the control group with LGK974 was set up (LGK974 group). In the PMSC+LGK974 group and LGK974 group, LGK974 was given by gavage (50 mg/kg/day).

### 2.2. Stem Cell Preparation

#### 2.2.1. The Methods of Isolation and Culture of PMSCs Refer to Published Literature

The isolation protocol for PMSCS was established from the previous study [14]. In a nut shell, fresh placentas were collected from normal, full-term (38–40 weeks gestation), healthy donors in compliance. Approval of the Independent Ethics Committee of the Shanghai Tenth People's Hospital was taken. Written informed consent was signed prior to the study. The umbilical cord blood was allowed to drain before the dissection of the placentas. All tissues would be examined to exclude virus infections and processed within 3 hours.

The harvested tissues were washed four times in PBS and then manually minced and digested with 0.1% collagenase IV (Sigma-Aldrich) at 37°C for 1 hr. The tissue was filtered twice through a cell strainer (Falcon 3078; BD Biosciences, San Jose, CA, USA) to eliminate undigested fragments. After the cells were centrifuged at 350 g for 10 min and red blood cells were lysed by red blood cell lysis buffer for 5 min, at 37°C, then the remaining cells were centrifuged at 300 g for 5 min. The cell pellets were resuspended in DMEM medium containing 10% FBS, 100 units/mL penicillin, 100 *μ*g/mL streptomycin, 2 mM L-glutamine, and 1% nonessential amino acids. The cells were cultured at 37°C under a 5% CO_2_ atmosphere for 4 days before the culture medium was first changed and a 70–80% cellular confluence was obtained [16].

### 2.3. Thermal Stimulation Response

The mice's thermal stimulation response was measured with plantar analgesia tester (Taimeng, Chengdu, China). The mice were placed on a 3 mm thick glass, and the heat stimuli were exposed below the glass. Thermal withdrawal latency was the elapsed time to withdraw the paw. Each group was averaged for 5 measurements, and each time interval was 10 minutes or longer. In order to avoid tissue damage, a 60 s cutoff time was set [17].

### 2.4. Western Blot Analysis

All samples were taken from the sciatic nerve. First isolate the sciatic nerve and then lyse by radioimmunoprecipitation assay buffer supplemented contain protease inhibitor cocktail (Beyotime Technology; China). Every sample loaded a total protein of 15 *μ*g and separated into 8% SDS polyacrylamide gels. The proteins were then transferred to polyvinylidene difluoride membrane, blocked with Tris-buffered saline 5% nonfat dry milk. Then, membranes were incubated with the primary antibodies at 4°C overnight. After being washed with PBS 3 times, membranes were cultured with secondary antibody for 1 h at room temperature. Proteins were detected using the ECL detection kit (Epizyme, China). Anti-*β*-actin, anti-Bax, anti-*β*-catenin and anti-DVL1 antibodies were used (Abcam, Cambridge, UK). The signal intensities were quantified by ImageJ.

### 2.5. Immunofluorescence

Immunofluorescence assay for PGP9.5 and S100*β* levels was performed. Tissue was deparaffinized in xylene and rehydrated in ethanol and then blocked with 5% bovine albumin for 30 min and incubated with primary antibody (Thermo Fisher Scientific, USA) for 1.5 h at room temperature. After having washed with PBS, incubation was done with a secondary antibody (Beyotime, China), counterstained with DAPI 1 : 2000, and eventually observed under the fluorescence microscope (Nikon, Japan).

### 2.6. Electron Microscopic Examination

After having washed with PBS, the sciatic nerve was fixed with 2.5% glutaraldehyde for 4 hours and postfixed with 1% OsO_4_ for 2 h both in phosphate buffer. Subsequently, dehydration of the sciatic nerve was performed in the graded ethanol series (50, 70, 80, 90, 95, and 100%). Lastly, sciatic nerves were infiltrated with acetone and EPON 812 (90529774, SPI-Chem, PA, USA), 1 : 1 for 2 h, 2 : 1 overnight, and absolute EPON 812 overnight. Images were captured and analyzed by TEM (HT7700, Hitachi, Japan).

### 2.7. Statistical Analysis

LSD (least significant difference) *t*-test variance analysis and student *t*-test analyzed the differences between groups. *P* ≤ 0.05 were considered statistically significant. Fundamental analyses were accomplished by SPSS 13.0, and all data were expressed as the mean ± SEM.

## 3. Results

### 3.1. The Nerve Has Been Damaged before the Experiment

**Figure 2:**
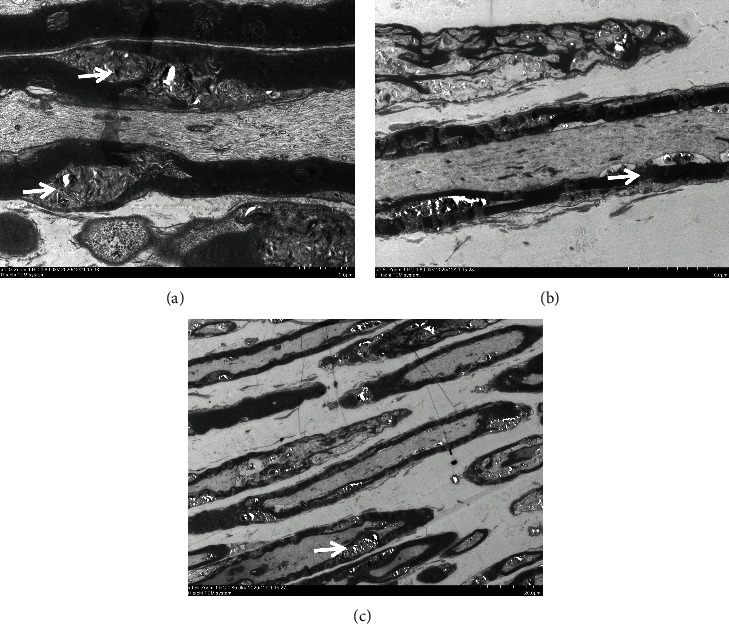
Nerve damage before treatment (the white arrow). Myelin sheath damage was observed at different magnifications, and this change was more obvious at higher magnifications (scale is in the lower right corner).

Before treatment, the sciatic nerves of some of the mice were removed and the damage was observed under an electron microscope. At different magnifications, the damage of myelin sheath of these nerves can be seen (Figure 2).

### 3.2. Effects of PMSCs on Blood Glucose and Body Weight

**Figure 3:**
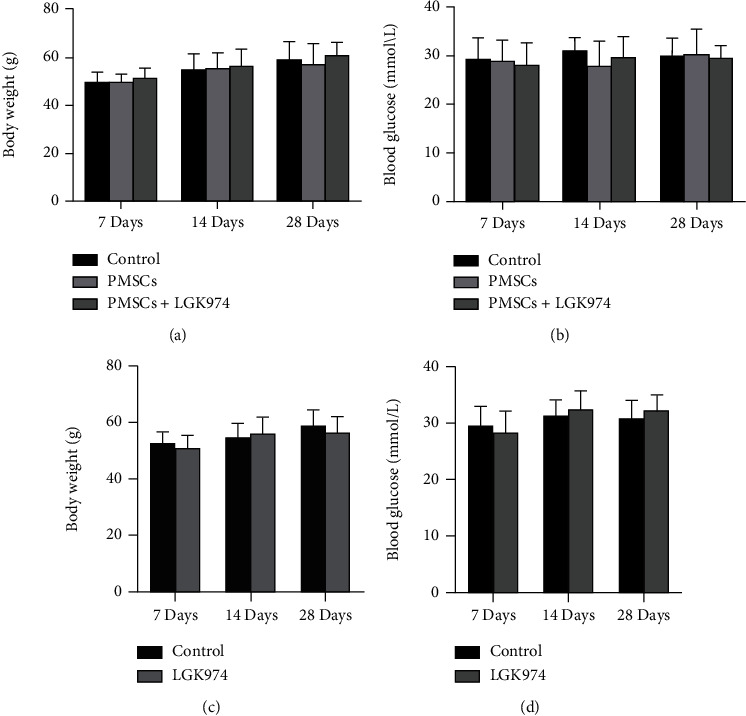
Blood glucose and body weight: (a, c) blood glucose (mmol/L); (b, d) body weight (g). No significant difference in body weight and blood glucose was noted between the control group, PMSC group, and PMSC+LGK974 group at 7, 14, and 28 days. No significant difference between the control group and LGK974 group at 7, 14, and 28 days.

The effects of PMSCs on blood glucose and body weight were observed after 7, 14, and 28 days of treatment. No significant difference in blood glucose and body weight was noted among those groups at 7, 14, and 28 days after treatment (Figure 3).

### 3.3. The Effect of PMSCs on the Thermal Stimulation Response

**Figure 4:**
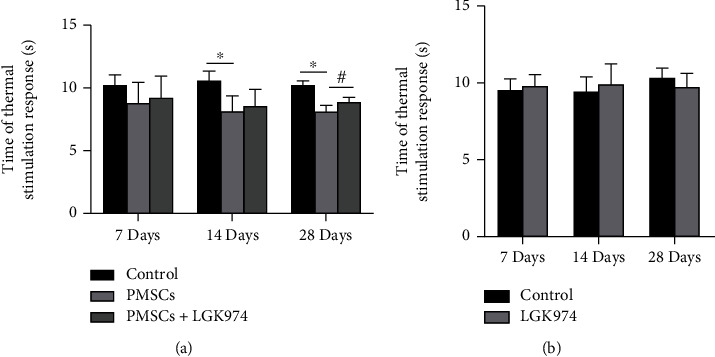
Time of thermal stimulation response(s). There was no difference in time of thermal stimulation response in three groups at 7 days. PMSC treatment significantly improved thermal stimulus response at 14 and 28 days compared with the control group. LGK974 inhibitor significantly decreased the therapeutic effect of PMSCs. ^∗^PMSC group vs. the control group, *P* < 0.05; ^#^PMSC group vs. PMSC+LGK974 group, *P* < 0.05 (a). No significant difference between control group and LGK974 group at 7, 14, and 28 days (b).

Thermal stimulation reaction is a common method for evaluating peripheral neuropathy in diabetic mice. To understand the effect of PMSCs on diabetic neuropathy, we tested thermal stimulation reactions on 7, 14, and 28 days. The thermal stimulation reaction in the PMSC group was faster than in the control group at 7, 14, and 28 days. This result suggested that PMSC therapy improved the sensitivity of peripheral nerves to heat stimulation and improved the protective response of diabetic mice. After the application of LGK974 inhibitor, the therapeutic effect of PMSCs was significantly inhibited. No significant difference in the time of thermal stimulation response was noted among those groups at 7, 14, and 28 days after treatment (Figure 4).

### 3.4. PMSCs Promoted the Expression of PGP9.5

**Figure 5:**
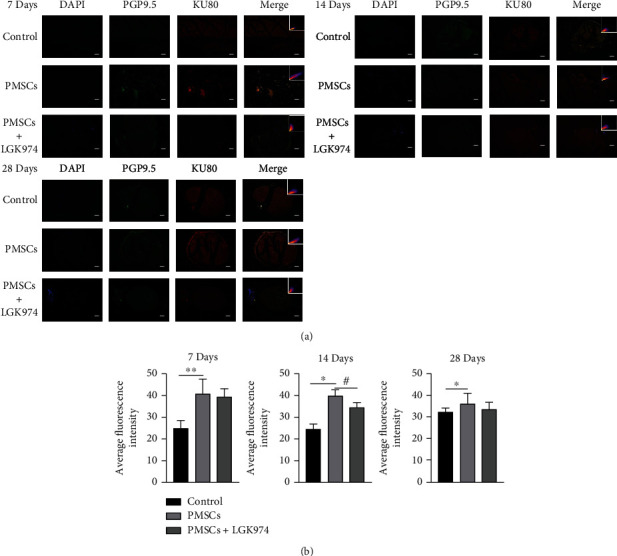
Immunofluorescence imaging of PGP9.5 and Ku80 in the sciatic nerve. (a) PGP9.5 was highly expressed in the PMSC group but lower in the control group at 7, 14, and 28 days. Contrarily, the upregulation of PGP9.5 by PMSCs was significantly inhibited by LGK974, and there was statistically significant difference at 14 days. The results demonstrated that KU80 was expressed in sciatic nerve. (b) Quantitative measurements were expressed as mean fluorescence intensity. Data were mean ± SEM. *n* = 3; ^∗^PMSC group vs. control group, P<0.05; ^#^PMSC group vs. PMSC+LGK974 group, *P* < 0.05. The scale bar: 50 *μ*m.

PGP9.5 is a neuron marker, which is used as a marker for neurogenesis. We used immunofluorescence to detect the expression PGP9.5 in the sciatic nerve. The results showed that the treatment of PMSCs increased the expression of PGP9.5 (Figures 5(a) and 5(b)), indicating that PMSCs probably promoted neurogenesis. However, the effect was inhibited by LGK974. The expression of PGP9.5 was lower than that in the PMSC group at 14 days (Figures 5(a) and 5(b)). To understand the association between PMSCs and PGP9.5, we labeled PGSCs with KU80 which is a species-specific human protein. Fluorescence colocalization showed that the highlighted region of PGP9.5 was consistent with the position of PMSCs; Pearson's correlation coefficients are 0.75 ± 0.17 and 0.74 ± 0.24 in PMSC group at 7 days and 14 days.

### 3.5. PMSCs Increased the Expression of S100*β*

**Figure 6:**
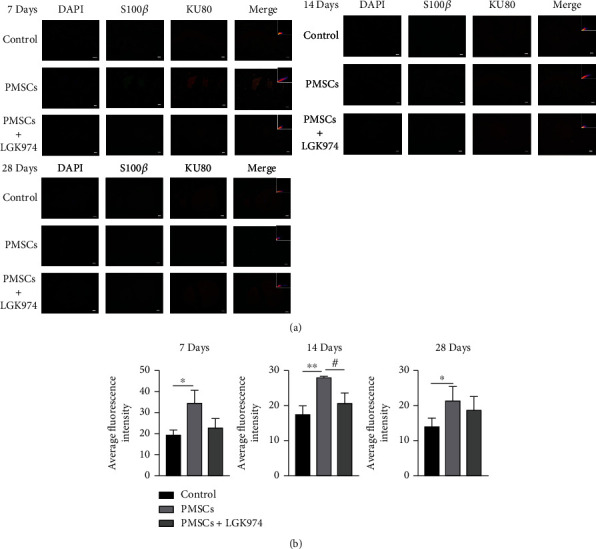
Relationship between Schwann cells and PMSCs. (a) The results of immunofluorescence showed that compared with the control group S100*β* in the PMSC group was highly expressed at 7, 14, and 28 days after treatment (*P* < 0.05); the expression of S100*β* in the LGK974 treatment group was significantly lower than that in PMSC group on 14 days. Fluorescence colocalization showed that the expression of S100*β* and KU80 overlapped in the same position after PMSC treatment. (b) Quantitative measurements were expressed as mean fluorescence intensity. Data were mean ± SEM. *n* = 3 per each group. ^∗^PMSC group vs. control group, *P* < 0.05; ^#^PMSC group vs. PMSC+LGK974 group, *P* < 0.05. The scale bar: 50 *μ*m.

S100*β* is a marker of Schwann cells. We tested the expression of S100*β* to observe the effect of PMSCs on Schwann cells. The results exhibited significant upregulation in the expression of S100*β* in PMSC group compared with the control group. However, the expression of S100*β* was significantly inhibited in PMSC+LGK974 group on 14 days (Figures 6(a) and 6(b)). These results suggested that PMSCs are likely to exert therapeutic effects through Schwann cells.

To further understand the association between PMSCs and Schwann cells, we labeled PMSCs using Ku80. The positions of S100*β* and KU80 were consistent (Figures 6(a) and 6(b)), indicating a spatial association between S100*β* and Ku80; Pearson's correlation coefficients are 0.82 ± 0.15 and 0.67 ± 0.13 in the PMSC group at 7 days and 14 days.

### 3.6. PMSCs Promoted the Repair of Nerve Myelin Sheath

**Figure 7:**
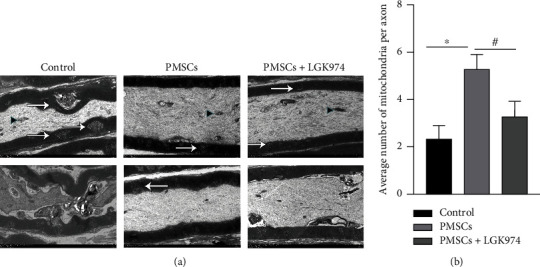
After transplantation, the structure of sciatic nerve was observed under electron microscope at 28 days. (a) There were more mitochondria in the PMSC group than those in the control group and PMSC+LGK974 group (*P* < 0.05). The myelin sheath of the PMSC group was more complete. Black triangles indicate mitochondria, and white arrows indicate lesions. (b) Number of mitochondria per axon. Data were mean ± SEM. *n* = 3 independent experiment; ^∗^PMSC group vs. control group, *P* < 0.05; ^#^PMSC group vs. PMSC+LGK974 group, *P* < 0.05. The scale bar: 2 *μ*m.

To understand the influence of PMSCs on the neural structure, the ultrastructure of myelin was observed by transmission electron microscopy. The sciatic nerve in the control group showed demyelinating injury with onion globules along the edge of the myelin sheath and myelinated axon (arrow). After the treatment of PMSCs, the sciatic nerve's myelin sheath was uniform, dense, and complete (Figure 7(a)). The number of mitochondria in axons in the PMSC group was also comparatively higher than that in the control group (Figures 7(a) and 7(b)). Since mitochondria are organelles for energy metabolism, this meant that energy metabolism was more active in the PMSC group.

### 3.7. PMSCs Upregulating Wnt Signaling Pathway

**Figure 8:**
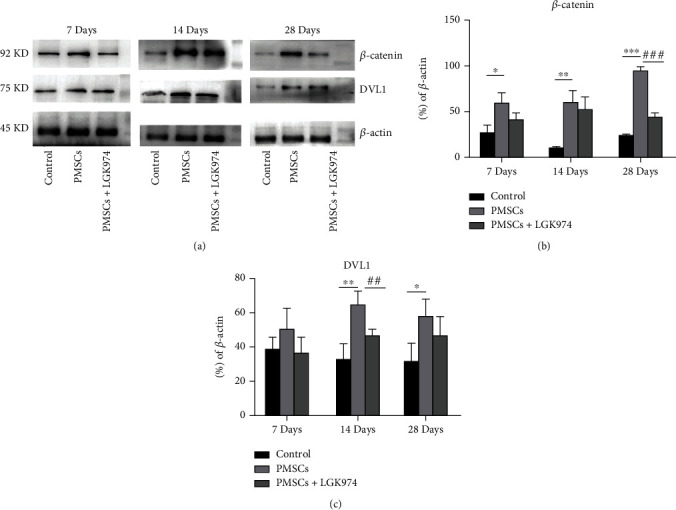
Effects of stem cell transplantation and Wnt signaling pathway inhibition on nerve repairing. (a) PMSC treatment activates *β*-catenin on 28 days. There was more increase in DVL1 in the PMSC group on 14 days. PMSCs decrease Bax on 7, 14, and 28 days. LGK974 inhibited *β*-catenin on 28 days and decreased DVL1 on 14 days. (b, c) Quantification of western blot bands of DVL1 and *β*-catenin was summarized. Data were expressed as the mean ± SEM. *n* = 3 independent experiment; ^∗^PMSC group vs. control group, *P* < 0.05; ^#^PMSC group vs. PMSC+LGK974 group, *P* < 0.05. All samples were taken from the sciatic nerve.

**Figure 9:**
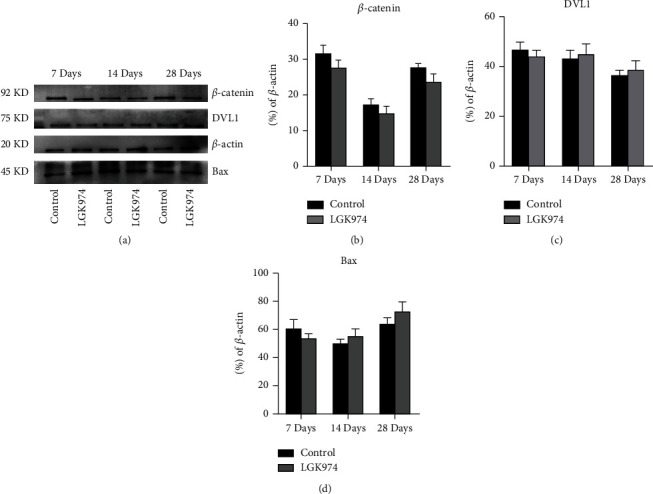
Effects of Wnt signaling pathway inhibition (LGK974) on nerve repairing. (a) The expression of *β*-catenin, DVL1, and Bax was not significantly increased or decreased. (b–d) Quantification of western blot bands of DVL1 and *β*-catenin was summarized. Data were expressed as the mean ± SEM. All samples were taken from the sciatic nerve.

In this study, we used Western blot to study the expression of essential proteins in the Wnt signaling pathway. *β*-Catenin was highly expressed after the treatment of PMSCs (Figures 8(a) and 8(b)), indicating that the Wnt signaling pathway was activated in PMSC treatment group. DVL1, which was strongly associated with nerve repair, began to increase significantly in 14 days (Figures 8(a) and 8(c)). The effect of LGK974 on *β*-catenin and DVL1 further verified the role of Wnt signaling pathway (Figures 8(a)–8(c)). These results suggested that PMSCs promoted nerve repair by upregulating the Wnt signaling pathway, especially on day 14. And LGK974 alone without PMSC transplantation had no significant effect on *β*-catenin and DVL1 (Figures 9(a)–9(c)).

### 3.8. PMSCs Inhibiting Expression of Bax

**Figure 10:**
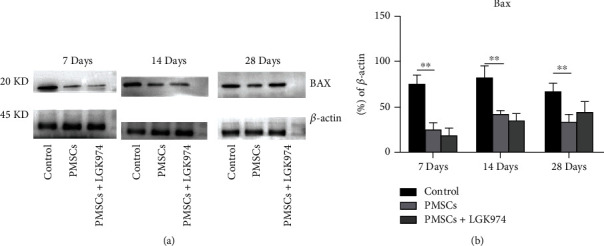
Effects of stem cell transplantation on Bax. (a) Bax decreased in PMSC group on 28 days. LGK974 had no significant effect on Bax. (b) Quantification of the expression of Bax was summarized. Data were expressed as the mean ± SEM. *n* = 3 independent experiment; ^∗^PMSC group vs. control group, *P* < 0.05. All samples were taken from the sciatic nerve.

The Bax is a highly conserved proapoptotic protein, and the expression of Bax in the PMSC group was lower compared with that in the control group (Figures 10(a) and 10(b)), indicating a weakening of apoptosis. Similarly, LGK974 itself had no significant effect on apoptosis, especially the apoptosis associated with Bax (Figures 9(a) and 9(d)).

## 4. Discussion

Diabetic neuropathy is considered one of the most detrimental chronic complications of diabetes. Mesenchymal stem cell transplant may be a novel strategy for diabetic neuropathy therapy, but its molecular mechanism is still unclear. The results of this study demonstrated that PMSCs not only improved the thermal response of nerve function but also promoted demyelination repair. The mechanism is related to the up-regulation of the expression of DVL1 and *β*-catenin. Pain and temperature sensation is a protective feeling, which protects the body from unnecessary damage, such as scalding. The response to thermal stimulation is a manifestation of protective reflexes. PMSC treatment significantly shortened the time of heat pain response in diabetic neuropathic mice. Resham and Sharma [13] confirmed PMSCs reduced the pain symptoms of diabetic neuropathic patients. He et al. [18] found PMSCs improved the phenomenon of slow response to mechanical stimulation pain in mice. Consistent with our results, PMSC therapy improved the symptoms of diabetic peripheral neuropathy.

Both rejection and survival rate after transplantation are more concerning to researchers. There was no obvious rejection in this PMSC treatment, and no inflammatory reaction was observed during the experiment, which was consistent with other experimental results [19]. In our previous experiment, the survival rate of PMSCs was about 5% 24 hours after transplantation [16]. The survival rate of this transplantation was not high (Figures 5 and 6), but they still played a role. In addition, this study showed that PMSCs promoted nerve myelin repair in diabetic mice. Diabetic neuropathy is characterized by axonal atrophy, nerve demyelination, and diminished rejuvenation of sensory nerve fibers of peripheral nerves [20]. PMSCs significantly upregulated the neurospecific protein PGP9.5, suggesting possible nerve regeneration. The results of electron microscopy showed that PMSC treatment reduced the area of myelin damage and improved the integrity of myelin sheath. Ku80 is a specific marker of human protein, so we used it to show the homing of stem cells. We found that PGP9.5 was correlated with Ku80 in fluorescence in colocalization, which indicated PMSCs probably actively home to the site of injured myelin nerve. Bax is a highly conserved Pro apoptotic protein. In some studies of nerve injury, the increase of Bax expression is considered to be related to nerve cell apoptosis [21]. In our experiment, the decrease of Bax is synchronized with the improvement of symptoms, which suggests that reducing the apoptosis of original nerve cells may also be one of the reasons for the improvement of symptoms, but this needs more evidence.

It is well established that Schwann cells are considered to be principal cells involved in nerve repair. Schwann cells lost contact with axons after nerve injury, which were activated, differentiated, entered the repair state, secreted nutrients and cytokines, and promoted axon repair of neurons [22]. Gonçalves et al. [23] found that Schwann cells played an indispensable role in axonal repair. Jessen and Mirsky [22] observed that Schwann cells in an active state were absent near neurons, leading to inhibiting repair after injury. We hence confirmed that the PMSCs transplant significantly augmented the expression of S100*β*, indicating that PMSC treatment of diabetic neuropathy might be related to Schwann cells. We also noted correlation between the positions of S100*β* and Belfiore L, suggesting that the therapeutic mechanism of PMSCs might also be related to Schwann cells [24]. However, the specific role and mechanism still needs further research and elaboration.

According to numerous studies, the Wnt signaling pathway is involved in nerve damage repair [25]. In the Wnt pathway, Wnt receptors and Wnt ligands are located on cell membrane and are the point of action of most Wnt pathway inhibitors [26–28]. *β*-Catenin and GSK3-*β* considered to be key proteins in the repair of damage, and their expression levels are greatly affected by the inhibitors [29]. DVL1 was believed to play a key role in central nervous system repair [30]. In this study, it was found that PMSCs improved neuropathy in diabetic mice, with the increase of *β*-catenin and DVL1. However, once the Wnt signaling pathway was inhibited, the therapeutic effect of PMSCs was greatly reduced, suggesting that the effect of PMSCs was at least partially related to the Wnt signaling pathway. BMSC alleviated diabetic peripheral neuropathy by increasing GSK-3 *β* and *β*-catenin, which is related to Schwann cells [31]. All the results confirmed that in diabetic neuropathy therapy of stem cell transplantation, the effect was related to the upregulation of the Wnt signaling pathway.

## 5. Conclusion

Placental derived mesenchymal stem cell transplantation can be homing the injured nerve and promote the repair of the injured nerve by upregulating the Wnt signaling pathway in treating diabetic neuropathy. The entire experiment has resulted in providing an innovative idea in the arena of diabetic neuropathic therapy.

## Data Availability

The datasets used and/or analyzed during the current study are available from the corresponding authors upon reasonable request.
